# Genomics reveal the origins and current structure of a genetically depauperate freshwater species in its introduced Alaskan range

**DOI:** 10.1111/eva.13556

**Published:** 2023-05-12

**Authors:** Matthew A. Campbell, Matthew C. Hale, Chase S. Jalbert, Kristine Dunker, Adam J. Sepulveda, J. Andrés López, Jeffrey A. Falke, Peter A. H. Westley

**Affiliations:** ^1^ University of Alaska Museum Fairbanks Alaska USA; ^2^ Department of Biology Texas Christian University Fort Worth Texas USA; ^3^ College of Fisheries and Ocean Sciences University of Alaska Fairbanks Fairbanks Alaska USA; ^4^ Division of Sport Fish Alaska Department of Fish and Game Anchorage Alaska USA; ^5^ Northern Rocky Mountain Science Center U.S. Geological Survey Bozeman Montana USA; ^6^ Alaska Cooperative Fish and Wildlife Research Unit, U.S. Geological Survey University of Alaska Fairbanks Fairbanks Alaska USA; ^7^ Present address: Division of Commercial Fisheries Alaska Department of Fish and Game Anchorage Alaska USA

**Keywords:** dispersal, *Esox lucius*, gene flow, invasive species, northern pike, population genetics

## Abstract

Invasive species are a major threat to global biodiversity, yet also represent large‐scale unplanned ecological and evolutionary experiments to address fundamental questions in nature. Here we analyzed both native and invasive populations of predatory northern pike (*Esox lucius*) to characterize landscape genetic variation, determine the most likely origins of introduced populations, and investigate a presumably postglacial population from Southeast Alaska of unclear provenance. Using a set of 4329 SNPs from 351 individual Alaskan northern pike representing the most widespread geographic sampling to date, our results confirm low levels of genetic diversity in native populations (average 𝝅 of 3.18 × 10^−4^) and even less in invasive populations (average 𝝅 of 2.68 × 10^−4^) consistent with bottleneck effects. Our analyses indicate that invasive northern pike likely came from multiple introductions from different native Alaskan populations and subsequently dispersed from original introduction sites. At the broadest scale, invasive populations appear to have been founded from two distinct regions of Alaska, indicative of two independent introduction events. Genetic admixture resulting from introductions from multiple source populations may have mitigated the negative effects associated with genetic bottlenecks in this species with naturally low levels of genetic diversity. Genomic signatures strongly suggest an excess of rare, population‐specific alleles, pointing to a small number of founding individuals in both native and introduced populations consistent with a species' life history of limited dispersal and gene flow. Lastly, the results strongly suggest that a small isolated population of pike, located in Southeast Alaska, is native in origin rather than stemming from a contemporary introduction event. Although theory predicts that lack of genetic variation may limit colonization success of novel environments, we detected no evidence that a lack of standing variation limited the success of this genetically depauperate apex predator.

## 1 INTRODUCTION

Biological invasions have been likened to one of the four horsemen of the ecological apocalypse (Diamond, 1984) and remain a leading cause of local and species‐wide extinctions (Clavero & Garciaberthou, 2005; Mooney & Cleland, 2001; Moyle & Leidy, 1992; Patankar et al., 2006; Ricciardi & Macisaac, 2010; Vitousek et al., 1996). Indeed, the legacy effects of species introductions that result in invasive hybridization (e.g., Muhlfeld et al., 2017) or novel predation by apex predators (e.g., Côté & Smith, 2018) create especially pernicious problems for natural resource management and conservation. Despite this adversity, biological invasions provide large‐scale, unplanned, replicated natural experiments to investigate fundamental ecological and evolutionary questions (Sax et al., 2007; Westley, 2011). Here we apply modern genomic tools to the invasion of a top freshwater predator, northern pike (*Esox lucius*: Esocidae; hereafter “pike”) to explore patterns of population divergence and to shed light on the origins of the invasion in Alaska, USA.

Pike is a circumpolar species of fish found throughout much of the Northern Hemisphere (e.g., Seeb et al., 1987). Despite their broad native range, pike are genetically depauperate with low diversity compared to other species of freshwater fish (Seeb et al., 1987; Senanan & Kapuscinski, 2000; Skog et al., 2014; Wennerström et al., 2018). This is likely due to their history as modern day populations were established after postglacial expansion from multiple geographically restricted refugia. Other fishes with similar ranges, and that presumably experienced similar bottlenecks, do not show the same reductions in genetic diversity (Miller & Senanan, 2003). Although it is well established that pike originated in Eurasia, how pike colonized and their subsequent dispersal in North America remain unknown (e.g., Johnson, 2019). There does appear to be genetic separation between native populations of Alaska and eastern North American pike, and a reduction in genetic diversity in eastern populations strongly suggests a Beringia origin of colonization into North America with subsequent expansion eastwards (Johnson, 2019; Senanan & Kapuscinski, 2000; Skog et al., 2014).

**FIGURE 1:**
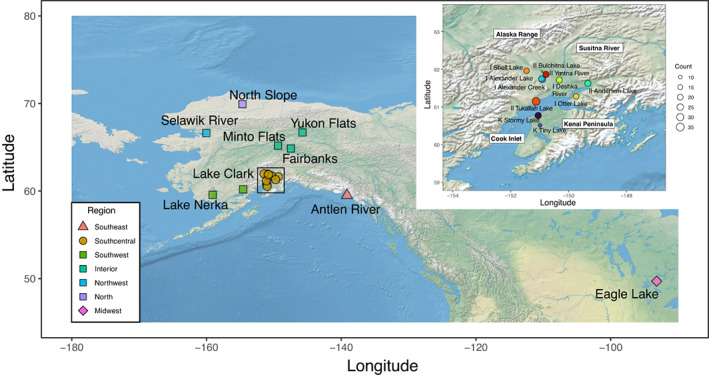
Sampling sites of pike sequenced in this study as detailed in Table 1. Pike are native to Alaska north and west of the Alaska Range indicated by squares, except for a putative relictual population in Southeast Alaska represented by Antlen River in this study indicated by a triangle (Morrow, 1980). Sampling sites in Southcentral Alaska are bounded by a box and indicated by circles in the main figure are considered introduced (Dunker et al., 2020). The inset shows Southcentral Alaska sampling sites with the size of points scaled to sample size. Sample site names are prefaced with labels corresponding to genetic grouping as explained in the text: I – Southcentral I; II – Southcentral II, K – Kenai Peninsula. Eagle Lake (diamond) is a geographically distant site from Alaska with a pike population representative of non‐Beringian pike.

Introductions of pike outside their native range have increased worldwide (Johnson et al., 2009; McMahon & Bennett, 1996). As a prized sportfish and common target of aquaculture enhancement, pike introductions are the product of natural and human‐assisted movements (Dunker et al., 2018). In Alaska, pike are an ecologically and culturally important native fish species north and west of the Alaska Range, but do not occur naturally south and east of the Alaska Range mountains (Figure 1). A possible exception—that has not been evaluated genetically—are populations of pike found in the Antlen River near Yakutat, Alaska (Morrow, 1980). The origins of these pike, relative to other native and introduced Alaskan populations, are unclear and genetic studies may help elucidate their origins. Elsewhere, south and east of the Alaska Range in Alaska, pike occurrences are clearly the result of human‐mediated introductions. Although the history of the introduced population is relatively unknown, records suggest that in the 1950s, a floatplane pilot transported pike from Minto Flats AK, USA (i.e., their native range), and released them into Bulchitna Lake (Matanuska‐Susitna Borough, AK, USA; Dunker et al., 2020). This illegal stocking event is the first known instance of pike in the Matanuska‐Susitna basin, although prior introductions may have occurred. Since then multiple stocking events have occurred throughout Southcentral Alaska and in lakes on the Kenai Peninsula, Alaska, until at least the early 2000s (Dunker et al., 2018; Haught & von Hippel, 2011). Continued identification of newly established populations associated with human‐assisted introductions and subsequent range expansion of non‐native populations indicates that this invasion is an ongoing process and that many currently un‐invaded sites remain vulnerable to colonization (Jalbert et al., 2021).

Despite widespread concern about the impacts of pike, particularly as predators of native Pacific salmonids (*Oncorhynchus* spp.), virtually nothing is known about the genetic diversity of Alaskan pike (although see Seeb et al., 1987; Skog et al., 2014; Wooller et al., 2015). We used genome‐wide RAD‐seq data approaches from pike from both native and introduced populations in Alaska to illuminate whether the origins of introductions were likely the result of single‐source or multiple introductory events. Specifically, our objectives were (1) to characterize the level of genetic variation in both invasive and native populations of Alaska pike, (2) to determine the most likely origin of invasive populations of pike, and (3) to clarify the origins of a presumed postglacial relict population of pike in the Antlen River near Yakutat in the context of our findings regarding known invasive and known native pike populations.

## 2 METHODS

### 2.1 Sampling, sequencing, and alignment of ddRAD dataset

Pike were collected from 20 sites that we treat as putative populations. Eleven sites are from within the species' non‐native range and nine from the native range (Figure 1 and Table 1). We selected invasive sites based on previous knowledge of human‐mediated introduction and to obtain representative geographic sampling across the introduced range. We selected native sites based on the likelihood of representing a source population for the invasion into Southcentral Alaska. These sites included the purported source of the Bulchitna Lake introduction (Minto Flats) and other locations with frequent floatplane and small plane transit relative to the introduced ranged (Southwest Alaska, Fairbanks) (Dunker et al., 2018; Jalbert et al., 2021).

**TABLE 1:** Sampling information of sites used for generation of RADseq data in this study.

Site	Latitude	Longitude	Status	Est. invasion year	*N*	Collector	Invasive genetic cluster
Antlen River	59.492422	−139.15668	Native		25		
Yukon Flats			Native		13		
Minto Flats	65.157778	−149.37	Native		20		
Fairbanks	64.849445	−147.85054	Native		20	UAF	
Lake Clark	60.182356	−154.55104	Native		21	NPS	
Lake Nerka	59.556508	−159.0761	Native		22		
North Slope	69.900517	−154.6456	Native		5		
Selawik River	66.603893	−160.00694	Native		2		
Eagle Lake	49.7	−93.21667	Native		9		
Stormy Lake	60.773717	−151.05258	Introduced	1970	23	ADFG	Kenai
Tiny Lake	60.519884	−150.99299	Introduced	Unknown	10	ADFG	Kenai
Alexander Creek	61.736635	−150.90074	Introduced	1970	20	ADFG	Southcentral I
Alexander Lake	61.736635	−150.90074	Introduced	1960	25	ADFG	Southcentral I
Deshka River	61.71497	−150.32448	Introduced	1980	21	ADFG	Southcentral I
Otter Lake	61.29013	−149.74095	Introduced	2000	20	ADFG	Southcentral I
Shell Lake	61.959494	−151.45015	Introduced	1980	20	CIAA	Southcentral I
Anderson Lake	61.620435	−149.33847	Introduced	1990	23		Southcentral II
Bulchitna Lake	61.935431	−150.9205	Introduced	1960	7		Southcentral II
Tukallah Lake	61.145748	−151.12661	Introduced	Unknown	35		Southcentral II
Yentna River at Indian Creek	61.867559	−150.77996	Introduced	1990	23		Southcentral II

*Note*: If not collected by authors of study, the collector is given: Alaska Department of Fish and Game (ADFG), Cook Inlet Aquaculture Association (CIAA), National Parks Service (NPS), University of Alaska Fairbanks (UAF).

Pike were captured using a combination of gillnet, seine net, rotenone treatment, and angling methods. All pike captured in the invasive range and up to 50 pike from each native site were euthanized. Pectoral fin clip tissues were placed in reagent alcohol (95%) or a solution of dimethyl sulfoxide (DMSO), Ethylenediaminetetraacetic acid (EDTA), and salt for preservation (Seutin et al., 1991) immediately after collection. Sample collection and associated protocols were approved by the Alaska Department of Fish and Game (ADF&G) and the Institutional Animal Care and Use Committee (IACUC) and were collected under Fish Resource Permit number SF2017‐168 and IACUC protocol number 921163‐3.

### 2.2 Genomic DNA isolation and RADseq

Total genomic DNA was isolated from preserved tissue samples using the reagents and protocols of the Qiagen Gentra Puregene Tissue kit (QIAGEN Inc., Valencia, CA, USA) with a modification to the final elution buffer. Specifically, isolated genomic DNA was dissolved in 50 μL of low‐EDTA TE buffer (pH 8.0) to minimize potential inhibition of downstream reactions. Quantity and purity of DNA preparations were assessed through fluorometry, spectrophotometry, and electrophoresis. We used a double‐digest restriction‐site‐associated sequencing (ddRAD‐seq) approach (Peterson et al., 2012) to broadly characterize genetic variation. A sample size of up to 25 randomly selected individuals per site was based on previous studies that used ddRAD‐seq datasets to determine genetic diversity (Hale et al., 2012; Willing et al., 2012). Briefly, ≥200 ng of DNA from each sample was digested using the restriction enzyme combination *MspI* (C|CGG) and *EcoRI* (G|AATTC). Restriction digest products were used to build individually tagged libraries, which were then pooled, and size selected to a range of 200–500 base pairs (Blue Pippin, Sage Science) and sequenced on an Illumina HiSeq platform using 2 × 125 bp PE V4 chemistry. Library construction, sequencing, and demultiplexing were performed by GENEWIZ LLC (South Plainfield, NJ, USA) with a targeted 5 million paired reads per individual.

### 2.3 Sequence data quality assessment and alignment to pike genome

We used FASTQC version 0.11.7 (Andrews et al., 2010) to assess the quality of the sequence reads and MULTIQC version 1.5 (Ewels et al., 2016) to obtain a consolidated summary of individual FASTQC module reports. Sequences and individuals with more than 20% of the bases exhibiting a Phred quality score (i.e., base calling accuracy) less than Q30 were removed from downstream analyses.

Quality‐filtered reads were aligned to V3 of the pike genome (Eluc_V3, Rondeau et al., 2014) using bwa‐mem with default parameters (Li & Durbin, 2009). SAMtools V1.6 (Li et al., 2009) was used to convert alignments into sorted BAM files for subsequent analysis. Conversion of SAM files to sorted BAM files was completed using default parameters. Coverage of each sample was assessed with SAMtools depth and the samples within the bottom 10% of coverage were removed from all subsequent analyses to create an “all samples” dataset.

### 2.4 Population genomic analyses

Genotypes were called from sorted BAM files using ANGSD (v0.922; Korneliussen et al., 2014) using the following parameters: minMaf 0.05, minMapQ 20, minQ 20, and SNP_pval 1E‐6. Genotypes were further filtered to ensure that 80% of samples produced a supported genotype call. For F_ST_ analysis, genotype likelihoods were used to generate the folded frequency spectrum. The frequency spectrum was then used to calculate genome‐wide weighted F_ST_ values for all pairwise comparisons in populations consisting of more than 10 samples (*n* = 338, 15 sites). The site frequency spectrum was also used to generate Tajima's *D* and nucleotide diversity (𝝅) statistics for each population. Both Tajima's D and 𝝅 were estimated on a chromosome level and boxplots (using ggplot2) were created to show the variance between populations.

Admixture analysis was performed using NGSadmix (Skotte et al., 2013). Beagle files generated by ANGSD (minMaf 0.05, minQ 20, minMapQ 20) were used as input for NGSadmix. NGSadmix was run on two datasets: (1) all Alaska pike samples from sites with *n* > 6 individuals (*n* = 344, 17 sites) and (2) introduced samples from Southcentral Alaska (*n* = 225, 11 sites). Analyses were repeated with *K* set to varying numbers (range from 2 to 10 for the complete dataset and 2–5 for the Southcentral Alaska samples). Ancestry plots were then created in R (R Core Team, 2018) using both the ggplot2 and melt packages (Wickham, 2009). Optimum *K* values were inferred based on a combination of maximum likelihood scores and the observed relationship between populations.

To examine population structure evidenced by an alternative method to NGSAdmix, we applied Discriminant Analysis of Principal Components (DAPC) as implemented in the R package adegenet (Jombart, 2008; Jombart et al., 2010). Discriminant Analysis of Principal Components implements successive *k*‐means clustering for different values of *K* genetic clusters maximizing between group variance. For all Alaska pike samples from sites with *n* > 6 individuals (*n* = 344, 17 sites), we generated a called set of genotypes following the same parameters as the admixture analysis but also enforcing a posterior cutoff option (−postCuoff 0.95) with ANGSD and outputting the genotypes as a PLINK‐formatted file. Then we used PLINK (Purcell et al., 2007) to convert from PLINK to VCF format, using vcfR R package functions that read in a VCF file and convert to a genind object for use with adegenet within R (Knaus & Grunwald, 2017). We examined *K* = 2 genetic clusters to identify the largest division in the dataset and subsequently identified an optimal *K* genetic clusters. An optimal *K* genetic cluster of individuals was determined by successive *k‐*means clustering as implemented by the *find. clusters* function of adegenet and the optimal *K* selected through Bayesian information criterion (BIC). Subsequently, an undivided geographic grouping of introduced pike was investigated by a separate genotype calling and DAPC analysis using the same parameters and methods.

### 2.5 Phylogenetic analyses of Alaska pike

We examined the relationships of Alaska pike with a phylogenetic approach that utilized 358 samples from 20 sites (including Eagle Lake from Canada) collected and sequenced in this study (Table 1). These 20 sites included 11 introduced pike sites and 8 natural pike sites from within Alaska. Genotypes were generated as a PLINK‐formatted file with ANGSD (v0.922; Korneliussen et al., 2014). Quality control thresholds were set with minimum mapping and base quality of 20 (−minMapQ 20, −minQ 20), a posterior cutoff of 0.95 (−postCutoff 0.95), a SNP *p* – val (−SNP_pval 1e−6), as well as a minimum MAF of 0.05 (−minMaf 0.05). The PLINK file was converted to a VCF formatted file with PLINK v1.9 (Chang et al., 2015) and the +prune algorithm of BCFtools v 1.10.1 (bcftools +prune −l 0.9 −w 10,000) applied. The pruned SNPs were prepared for SNP‐based phylogenetic analysis by removing sites that would be considered invariant due to ambiguities and formatted as a PHYLIP file (https://github.com/btmartin721/raxml_ascbias/blob/master/ascbias.py, https://github.com/edgardomortiz/vcf2phylip). We then generated a consensus tree based on 1000 rapid bootstraps (−bb 1000) in IQ‐TREE v2.0‐rc1 (Minh et al., 2020). Concatenated phylogenetic inference does not model recent or ancient admixture so TreeMix (Pickrell & Pritchard, 2012) was applied to the same SNP alignment as the concatenated phylogenetic inference as conducted by IQ‐TREE to identify potential admixture events in a phylogenetic framework.

### 2.6 Origins of Antlen River northern pike near Yakutat

We examined the relationships of Antlen River pike to native pike collected from natural occurrences in northern North America to determine which lineage (Beringian or Eastern North American) Antlen River pike are most closely related to and to further investigate if these fish are an introduced population from elsewhere in Alaska. We filtered our ddRADseq dataset to sites presumed to be native (*n* = 9) and obtained additional geographic coverage with three additional sites by downloading whole‐genome resequencing data from the NCBI Sequence Read Archive. Whole‐genome‐resequencing data from Palmer Lake in Northern British Columbia (*n* = 3, SRS4199890, SRS4199909, SRS4199915), the Yukon River ~90 km NE of Whitehorse, Yukon Territory (SRS4199894, SRS4199895, SRS4199896), and Minto Flats, Alaska (SRS4199902, SRS4199903, SRS4199900). The whole‐genome resequencing data included a resampling of Minto Flats to verify intercompatibility of the data types (RADseq and WGS) and two locations from western North America that are geographically close to Antlen River and could be near relatives. Whole‐genome resequencing data were aligned to a reference following the methods applied to ddRADseq data.

Genotype calls were generated with ANGSD (v0.922; Korneliussen et al., 2014) as a PLINK‐formatted file with the following parameters: SNPs were required to be genotyped in 95% of individuals (−minInd 132), minimal mapping and base quality were set to 20 (−minMapQ 20, −minQ 20), and a posterior cutoff of 0.95 was used (−postCutoff 0.95). To reduce method and population‐specific SNPs, a minimum minor allele frequency of 0.30 was specified (−minMaf 0.30). The resulting PLINK‐formatted file was converted to a VCF file with PLINK v1.9 (Chang et al., 2015) and linked SNPs removed with the +prune algorithm of BCFtools v 1.10.1 (bcftools +prune −l 0.9 −w 10,000). We first examined relationships of pike through Principal Component (PC) analysis of the pruned data. The VCF file of pruned SNPs was imported into R and converted to a genind object with the read.vcfR and vcfR2genind functions of the vcfR package. The genind object was translated to a matrix of alleles and missing data filled with mean values with the tab function of the adegenet package on which PC analysis was conducted with the dudi.pca function of the ade4 library. The first three principal components were visualized with ggplot functions.

Phylogenetic analysis was conducted by first converting the pruned VCF file to PHYLIP format with vcf2phylip.py (https://github.com/edgardomortiz/vcf2phylip) and invariant sites removed with ascbias.py (https://github.com/btmartin721/raxml_ascbias). An input file for SVDQuartets was made by grouping samples by location and data type. A set of 1,000,000 random quartets was evaluated and 100 bootstrap replicates were undertaken to assess confidence in inferred relationships.

## 3 RESULTS

### 3.1 Data summary

We generated ddRAD‐seq data from 351 individuals from 19 sites across Alaska that included sites within the species native (eight sites, *n* = 83) and introduced ranges (11 sites, *n* = 268). In addition, we sequenced seven individuals from a single site in Western Canada yielding a total of 358 pike. The sample distribution is shown in Figure 1 and summarized in Table 1. The average number of reads mapped to the reference genome for each sample is 9,215,134 which, on average, was 95.44% of the total reads sequenced. Additional information about each individual sample, including the number of aligned reads to the reference genome, is presented in Table S1.

### 3.2 SNPs and genetic variability

**FIGURE 2:**
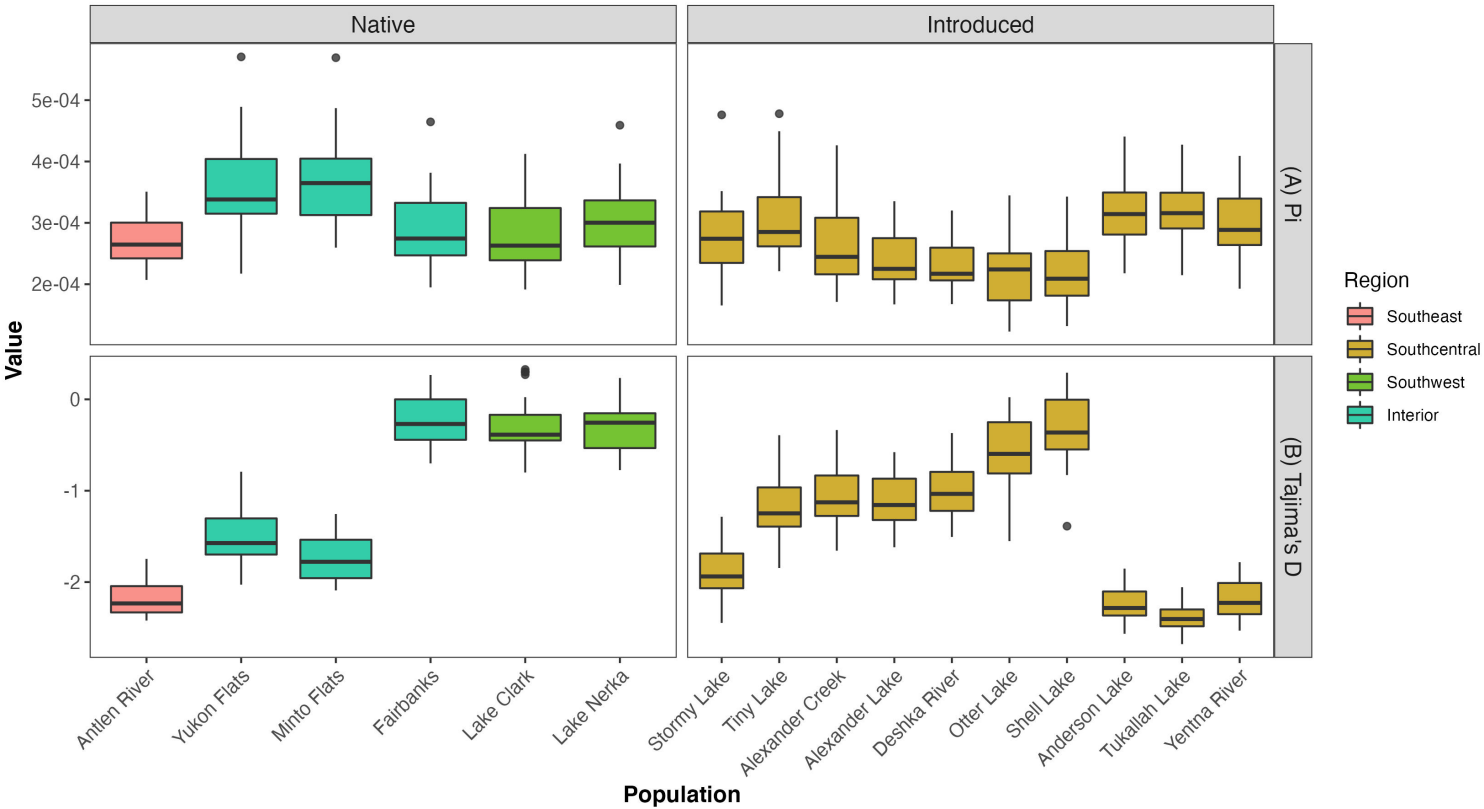
(a) Mean estimates of genetic diversity (Pi) calculated on a chromosome by chromosome approach. (b) Mean estimates of Tajima's D calculated on a chromosome by chromosome approach. Populations are colored to represent origin. Southcentral populations are representative of the introduced range of pike in Alaska.

From the Alaska pike samples (*n* = 351), we found a total of 4329 SNPs that had a minor allele frequency greater than 0.05 and were genotyped in at least 281 samples. Levels of nucleotide diversity were low in all populations and varied from 2.22 × 10^−4^ (Shell Lake; introduced) to 3.63 × 10^−4^ (Minto Flats; native) (Figure 2a). Nucleotide diversity was significantly higher in native populations (mean π = 3.18 × 10^−4^) compared to introduced populations (mean π = 2.68 × 10^−4^; *p* = 0.048) although several introduced populations (Anderson Lake and Tukallah Lake, Figure 1) had nucleotide diversity estimates similar to native populations. The number of private alleles also differed between native and introduced populations, with native pike populations having on average 891 private alleles (i.e., population specific alleles) compared to 198 for introduced populations (*p* = <0.001). Measurements of Tajima's D also showed a substantial difference between native and introduced populations, with introduced populations showing a more negative value indicative of population expansion following a major bottleneck (Figure 2b). Introduced populations had a lower Tajima's D value (mean = −1.42) than native populations (mean = −0.811), although this difference was not statistically significant.

### 3.3 Population structure and genetic differentiation

The introduced populations were split into three groups referred here as Southcentral I (Alexander Creek, Alexander Lake, Deshka River, Otter Lake and Shell Lake), Southcentral II (Bulchitna Lake, Anderson Lake, Tukallah Lake and Yentna River), and Kenai Peninsula (Stormy Lake and Tiny Lake) (Figure 1). These groupings were evident and consistent across analyses of population genetic structure and differentiation.

Pairwise estimates of F_ST_ varied widely between comparisons that ranged from 0.419 (between Antlen River and Otter lake) to 0 (−0.003; between Deshka River and Alexander Lake) (Table 2). F_ST_ values were far lower between introduced populations than between introduced and native populations, or between native populations. For example, pairwise comparisons within Southcentral I (Figure 1)—Alexander Lake, Alexander Creek, Deshka River, Otter Lake, and Shell Lake—produced an average pairwise F_ST_ of 0.029, suggesting minimal genetic differences among these introduced populations. Similarly, estimates of pairwise F_ST_ within Southcentral II (Figure 2)—Anderson Lake, Tukallah Lake, and Yentna River—produced an average F_ST_ of 0.016 suggesting limited divergence. The third introduced group, Kenai Peninsula (Figure 1), showed moderately high F_ST_ between both the Southcentral I and Southcentral II genetic groups (0.305 and 0.284, respectively). Lastly, comparisons between the Southcentral I and Southcentral II genetic groups of pike also produced moderately high F_ST_ values (0.148), suggesting separate origins for the three introduced groups.

**TABLE 2:** Pairwise global weighted F_ST_ estimates between populations with at least 10 sampled pike.

	Antlen River	Yukon flats	Minto flats	Fairbanks	Lake Clark	Lake Nerka	Stormy Lake	Tiny Lake	Alexander Creek	Alexander Lake	Deshka River	Otter Lake	Shell Lake	Anderson lake	Tukallah Lake
Antlen River															
Yukon Flats	0.202														
Minto Flats	0.165	0.074													
Fairbanks	0.311	0.194	0.201												
Lake Clark	0.371	0.269	0.278	0.194											
Lake Nerka	0.350	0.259	0.268	0.176	0.143										
Stormy Lake	0.356	0.264	0.267	0.288	0.224	0.199									
Tiny Lake	0.430	0.321	0.307	0.278	0.222	0.194	0.064								
Alexander Creek	0.401	0.323	0.324	0.239	0.245	0.211	0.297	0.305							
Alexander Lake	0.405	0.321	0.322	0.261	0.256	0.224	0.309	0.334	0.019						
Deshka River	0.414	0.327	0.328	0.261	0.255	0.229	0.316	0.313	0.019	−0.013					
Otter Lake	0.419	0.327	0.335	0.267	0.270	0.237	0.304	0.303	0.048	0.041	0.042				
Shell Lake	0.415	0.325	0.331	0.263	0.270	0.228	0.299	0.301	0.032	0.035	0.035	0.030			
Anderson Lake	0.271	0.211	0.203	0.350	0.327	0.295	0.284	0.357	0.144	0.144	0.147	0.168	0.152		
Tukallah Lake	0.259	0.205	0.197	0.339	0.329	0.301	0.286	0.355	0.134	0.134	0.145	0.165	0.152	0.022	
Yentna River	0.266	0.204	0.193	0.339	0.331	0.299	0.281	0.355	0.135	0.135	0.144	0.165	0.154	0.018	0.008

**FIGURE 3:**
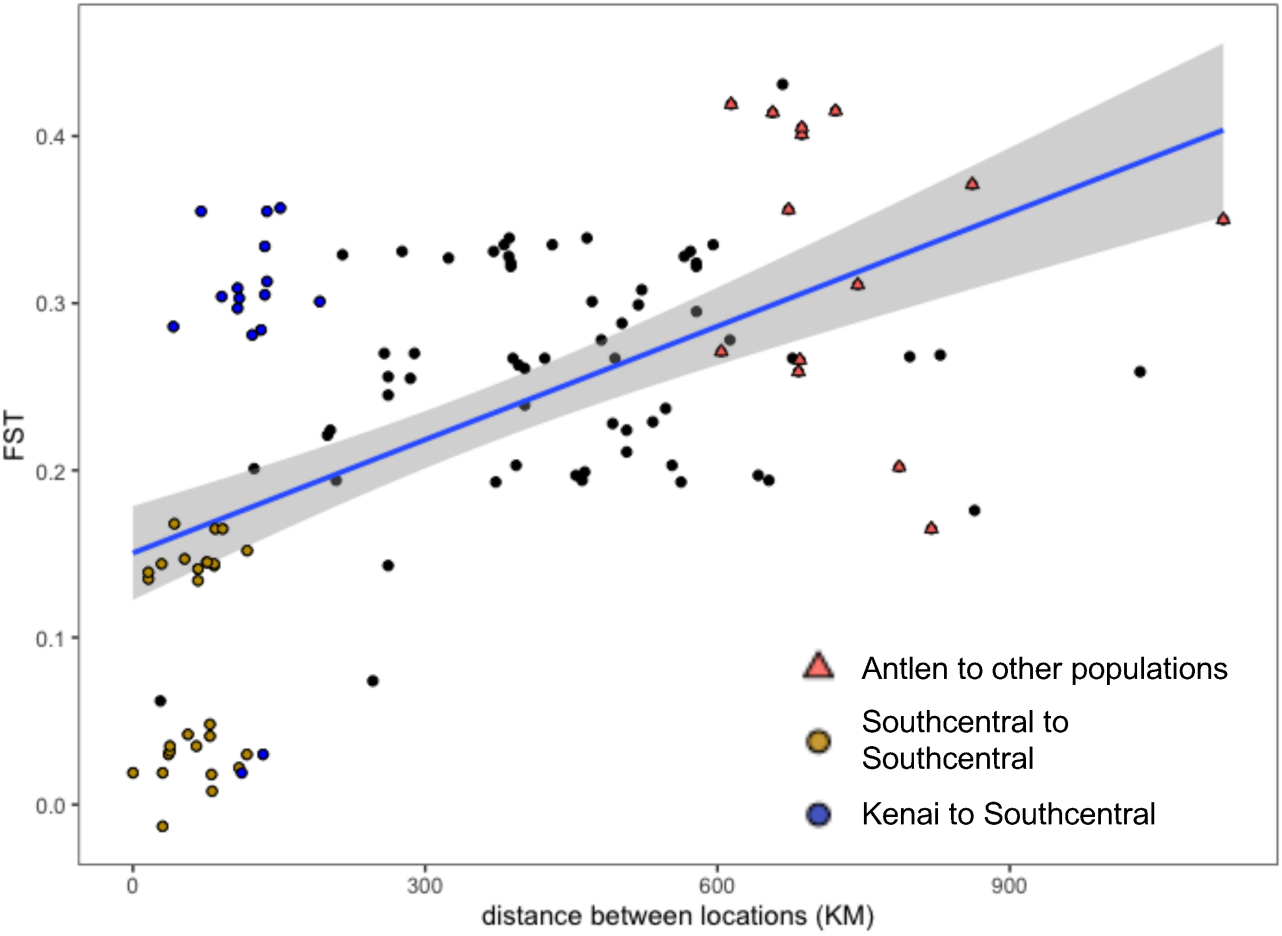
Scatter plot showing F_ST_ (FST) values as a function of mean distance between populations. Comparisons within the introduced range are indicated as circles with those between Stormy Lake (Kenai Peninsula) and all other locations colored in blue. Pairwise comparisons with Antlen River are indicated by red triangles.

Comparisons of F_ST_ between native populations were also high. For example, genome‐wide F_ST_ values between Fairbanks and Minto Flats were 0.201 and between Lake Clark and Lake Nerka 0.143. Lastly, genome‐wide F_ST_ results between the Antlen River were high in all comparisons regardless of the population's history (average F_ST_ for introduced populations = 0.356 and between Antlen and native populations = 0.280). Average F_ST_ showed a significant pattern of isolation by distance with a positive relationship between F_ST_ and the distance between populations (*R* ^2^ = 0.295, *F*_117_ = 49.036, and *p* < 0.001; see Figure 3). Several population comparisons were outliers in this analysis including comparisons with the Antlen River (higher F_ST_ values) and comparisons within both Southcentral groups (lower F_ST_ values).

**FIGURE 4:**
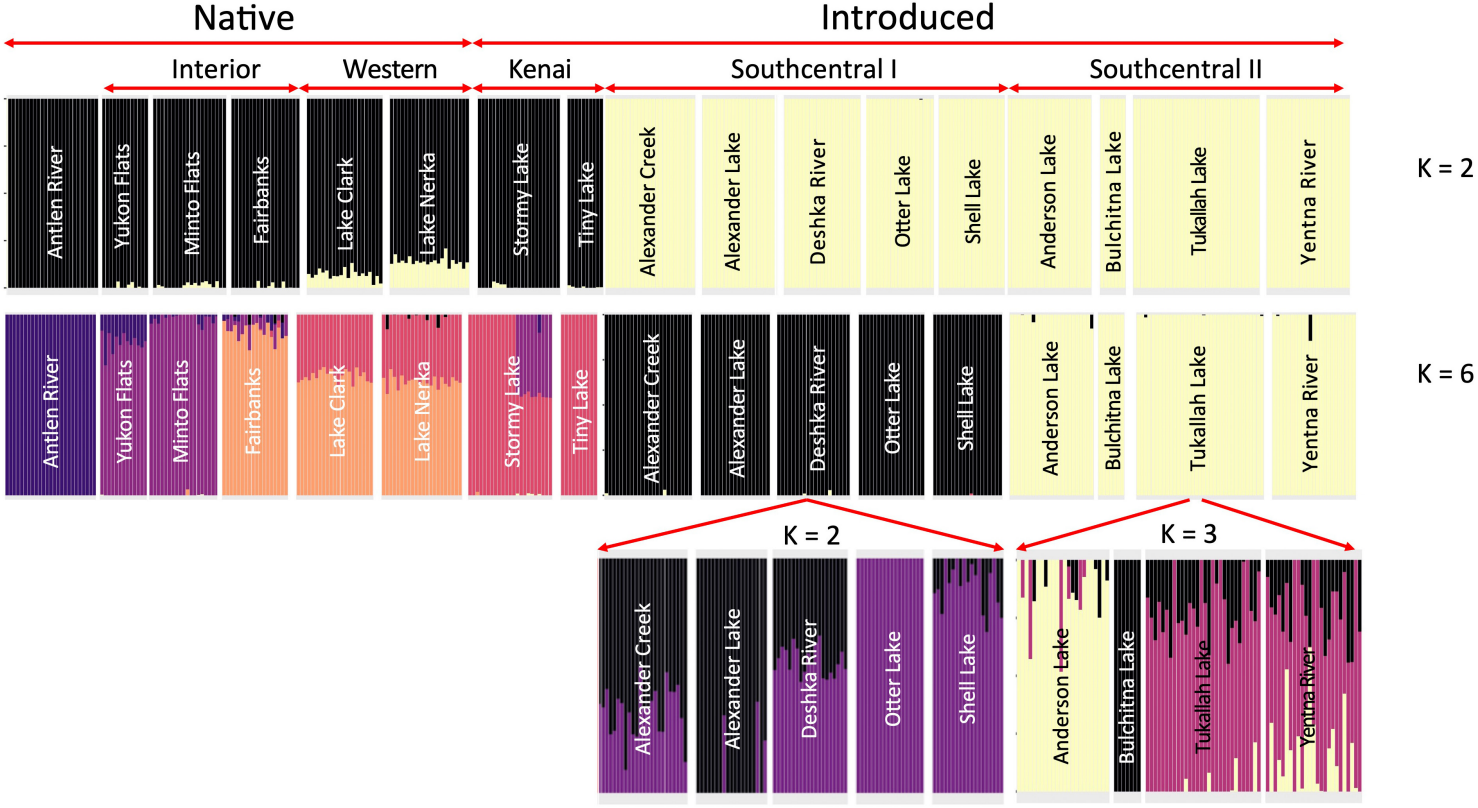
Admixture plots generated by NGSadmix of Alaska pike at *K* = 2 and *K* = 6. Sampling locations are subdivided by status (introduced vs. native) and then by geography, that is, Interior, Western Alaska, Kenai peninsular, then other Southcentral locations. Note that the classification of genetic clusters as Southcentral I vs. Southcentral II was based on allele sharing rather than geography (see Figure 1). Introduced populations were further subdivided, based on allele sharing, into either two genetic clusters (Southcentral I) or three genetic clusters (Southcentral II). Values of *K* were chosen based on the lowest log‐likelihood as implemented in NGSadmix software within ANGSD.

Our structure analyses suggested similar patterns of relatedness between populations as our F_ST_ results. For the complete dataset, *K* = 5 was the most supported. However, we varied *K* from 2 to 10 and reported on the major differences between groupings produced by those *K* values. When *K* = 2, the samples split into native and introduced groups with limited allele sharing between them (Figure 4). The two native populations from Western Alaska (Lakes Clark and Nerka) shared alleles with the introduced group, but the structure plot suggested the amount was low (<15%). When *K* = 3, the two Western Alaska samples split from the other native populations with some allele sharing with the two introduced populations on the Kenai Peninsula (Tiny Lake and Stormy Lake, Figure 1). When *K* = 4, the introduced populations not on the Kenai Peninsula split into two groups referred to here as Southcentral I (Alexander Creek, Alexander Lake, Deshka River, Otter Lake and Shell Lake) and Southcentral II (Bulchitna Lake, Anderson Lake, Tyonek, and Indian Creek). There was some allele sharing with the Southcentral II group with two native populations (Yukon Flats and Minto Flats), which is suggestive of native origins for the Southcentral II group. Similarly, the Southcentral I introduced group has some allele sharing with Fairbanks and Lakes Nerka and Clark. When *K* = 5, the Antlen River samples split from the other native populations with some allele sharing with Yukon Flats and fish from the North Slope. Increasing the *K* value to 6 caused the two Kenai Peninsula populations to split from native pike from lakes in Western Alaska.

A major aim of this research was to address the origin and relationships of introduced populations of pike in Alaska. Therefore, we also focused our admixture analyses on the introduced populations. Here, the optimal *K* value was 3 and could be broadly grouped into a Southcentral I genetic cluster, a Southcentral II genetic cluster, and the two lakes on the Kenai Peninsula. Increasing the *K* values suggested some lakes contained a high level of admixture and might be indicative of stocking from multiple populations and/or gene flow through dispersal. For example, when *K* = 4 the Southcentral I group began to divide, with Otter Lake and Shell Lake differentiated from Alexander Lake, whereas Deshka River and Alexander Creek appeared to contain alleles from both non‐native Southcentral groups suggesting admixture. Lastly, restricting our admixture analysis to just the Southcentral I populations suggested admixture in both Alexander Creek and Deshka River from fish from Otter Lake and Alexander Lake.

### 3.4 Discriminant analysis of principal components

**FIGURE 5:**
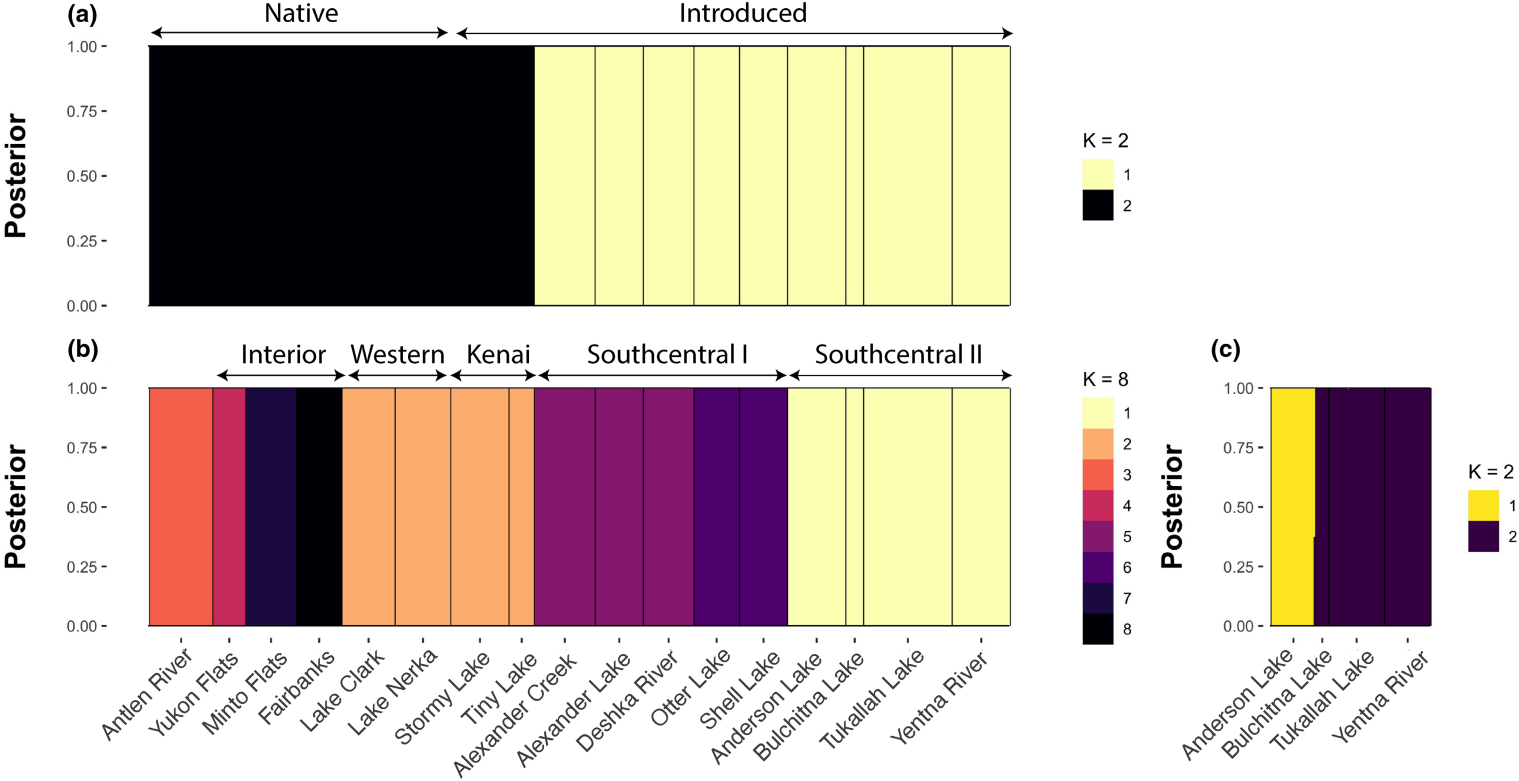
Discriminant Analysis of Principal Components (DAPC) of Alaska pike. (a) The largest division (*K* = 2) is shown. (b) The Bayesian information criterion (BIC) supported *K* = 8 genetic clusters of Alaska pike. (c) The Anderson Lake, Bulchitna Lake, Tukallah Lake, and Yentna River (Southcentral II) genetic cluster is further subdivided in a separate analysis of samples only in this cluster to *K* = 2.

**FIGURE 6:**
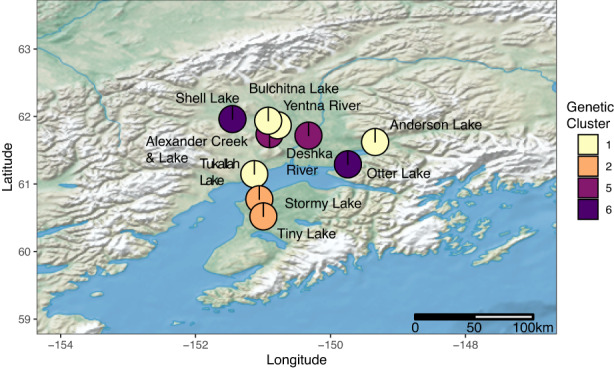
Geographic distribution of genetic clusters of introduced Alaskan pike in Southcentral Alaska. The averages of individuals for posterior assignment to eight genetic clusters (*K* = 8) from Discriminant Analysis of Principal Components in Figure 5b across collection sites are presented as pie charts. Four genetic clusters are present in Southcentral Alaska; genetic cluster 2 is shared between Stormy Lake and Tiny Lake (Kenai sites) and Lake Clark and Lake Nerka (Western Alaska). Other genetic clusters are found in introduced pike only.

A total of 345 individuals and 4860 loci were passed to DAPC after calling genotypes and removing sampling locations with few individuals (Selawik River, North Slope and Eagle Lake). When *K* = 2 genetic clusters is specified, the two observed clusters are composed of (1) native populations and Kenai Peninsula introduced pike and (2) all other introduced pike (Figure 5). Successive *k*‐means clustering indicated support for *K* = 8 genetic clusters with Antlen River, Yukon River, Minto Flats, and Fairbanks sampling locations identified as unique clusters. Groupings of sampling locations at *K* = 8 are Southwest Alaska and Kenai Peninsula pike (Lake Clark, Lake Nerka, Stormy Lake, Tiny Lake), and Southcentral I sampling locations are divided into two groups: one of Alexander Creek, Alexander Lake, and the Deshka River and a second of Otter Lake and Shell Lake. The genetic cluster present at *K* = 8 of Southcentral II (Anderson Lake, Bulchitna Lake, Tukallah Lake, and Yentna River) sampling locations was further examined by additional genotype calling for these fish (86 individuals, 4163 loci). This genetic cluster was subdivided by an optimal *K* of 2 splitting Anderson Lake from Bulchitna Lake, Tukallah Lake, and Yentna River sampling locations. The average membership in each genetic cluster for introduced pike sampling sites at *K* = 8 is plotted geographically in Figure 6.

### 3.5 Phylogenetic analyses of Alaska pike

**FIGURE 7:**
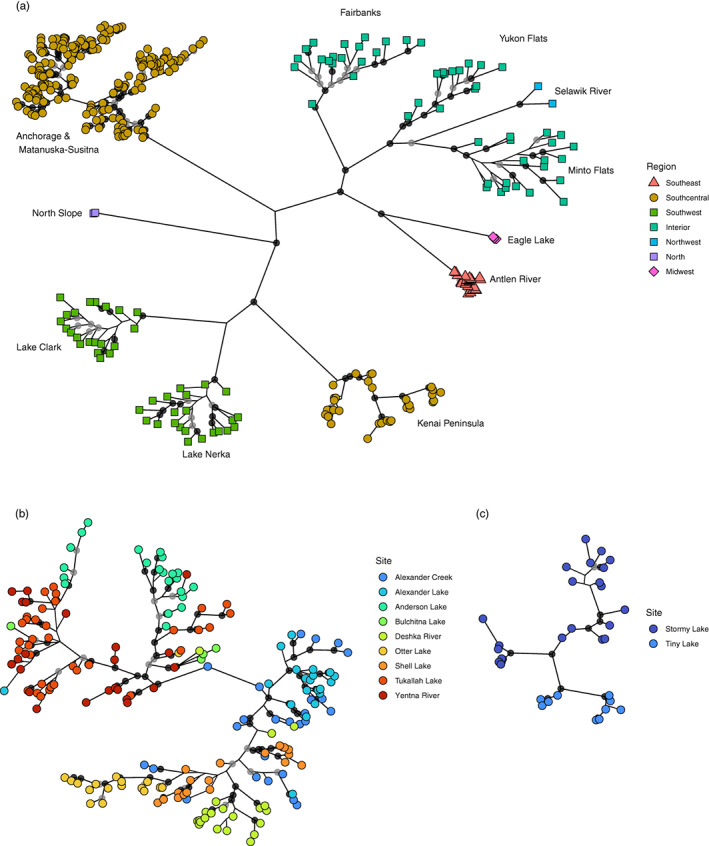
Individual phylogeny of pike sequenced in this study (Alaska and Eagle Lake, *n* = 358) with nodal support <75 not shown, ≥75 but less than 90 as light grey circles and ≥ 90 as black circles. Tip shapes and colors correspond to Figure 1. (a) The entire tree is pictured with colors of tips corresponding locations in Figure 1. (b) The subtree containing introduced pike from the Anchorage and Matanuska‐Susitna area is removed and colors are the same as the inset of Figure 1. (c) The Kenai Peninsula subtree is shown with colors matching the inset of Figure 1.

An initial 3614 SNPs were reduced to 907 with the consensus tree shown in Figure 7. A well‐supported clade comprised fish from Fairbanks, Yukon Flats, Selawik River, and Minto Flats sites, bootstrap support (BS) = 99% and monophyly of sites were highly supported (BS > 97%). Eagle Lake and Antlen River sites received strong (BS = 97%) support for the inferred placement and maximal (BS = 100%) support for monophyly of each site. The Anchorage area and Matanuska‐Susitna introduced pike were not clearly placed in the dataset in relation to other sampling locations but received maximal support for monophyly and were subdivided into two groups (Figure 7a,b). Southcentral I (Alexander Lake, Alexander Creek, Deshka River, Otter Lake and Shell Lake sites) had maximal support for monophyly. This Southcentral I group was nested within sites of Southcentral II (Anderson Lake, Bulchitna Lake, Tukallah Lake, and Yentna River). The Kenai Peninsula, Lake Clark, and Lake Nerka fish were highly supported in being placed together (BS = 100%); however, the branching patterns between these sites are not defined. Within the Kenai Peninsula, the Stormy Lake and Tiny Lake fish separated into two groups based on site (Figure 7c). TreeMix analysis produced topologies largely congruent with the concatenated phylogeny; notably a mixture event into the base of the Southcentral I grouping of pike is inferred (Figure S1).

### 3.6 Origins of Antlen River northern pike near Yakutat

**FIGURE 8:**
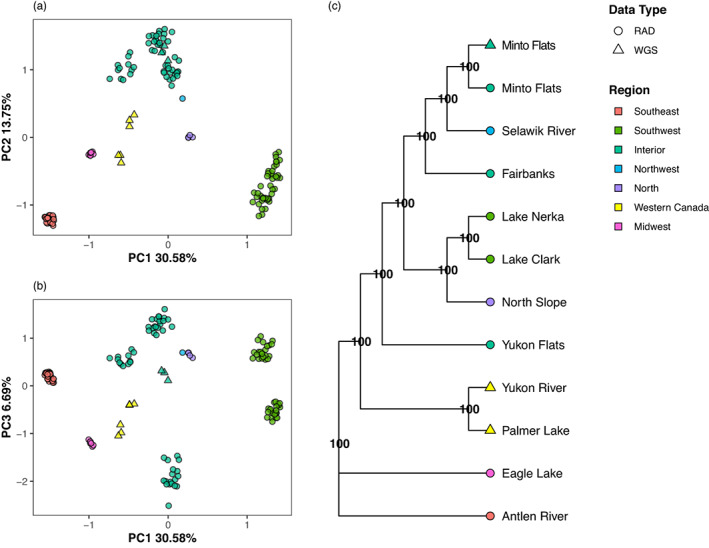
Placement of Antlen River pike. Additional sequence data from GenBank are analyzed with the RADseq from natural populations from this study. The whole‐genome sequence data (WGS) are indicated by triangles in the figure and RADseq by circles. The geographic origin of samples is indicated by color in the Region legend. (a) Principal component (PC) analysis of genetic variation showing the first two axes of variation. (b) Principal component analysis of genetic variation showing the first and third axes of variation. (c) Species tree from SVDQuartets. Nodal support is indicated on the tree.

Initially, from 139 samples from 12 presumably native pike populations, 837 SNPs met minimum MAF and other thresholds. These populations include eight sites in Alaska with RADseq data covering the broad natural range of pike in Alaska, a geographically distant site of native pike in Canada (Eagle Lake) and three locations from western North America from WGS data. Pruning reduced this number to 425 SNPs across native pike populations and the Antlen River pike. Plotting of PC 1 (30.58% of variance) and PC 2 (13.75% of variance) indicates Antlen River pike is a discrete group, separate from all other sampling locations examined (Figure 8a). Principal component 1 corresponded to the separation of Antlen River from all other pike, with Eagle Lake the closest on the PC 1 axis. The second PC separated Antlen and Southwest Alaska pike from Interior Alaska and the Yukon River pike. Principal Component 3 (6.69% of variance) largely separated Interior Alaska and Yukon River pike (Figure 8b).

For the construction of a species tree, 306 SNPs are available for use with SVDQuartets. Bootstrapping found maximal support for all inferred nodes. Antlen River and Eagle Lake were identified as most‐closely related (Figure 8c). Minto Flats origin pike of different data types were placed together in the tree and a close relationship indicated between North Slope and Southwest Alaska samples. Broadly, pike were distributed from East to West across the tree, the exception being Antlen River most closely related to the farthest East sampling location (Eagle Lake).

## 4 DISCUSSION

The introduction of non‐native northern pike in Alaska has had significant ecological impacts including the widespread reduction in the abundance of culturally, ecologically, and economically valuable Pacific salmon (Sepulveda et al., 2013). Despite this adversity, the introduction of pike in Alaska provides an excellent system to understand the genetic relationships between native and non‐native populations of colonizing species. Advances in modern genomic tools allow us to simultaneously illuminate the origins and population structure of non‐native populations. Our results yielded three major novel findings. First, we provide unequivocal evidence that pike in Southcentral Alaska are non‐native as all populations showed both low genetic diversity and a largely negative genome‐wide Tajima's D value, consistent with an excess of rare alleles resulting from population expansion following a severe bottleneck (Johnson, 2019; Wennerström et al., 2018). Second, we detected separate origins of introduction for non‐native populations in Susitna River/Yentna River region (i.e., Southcentral I and Southcentral II) compared to the Kenai Peninsula, strongly supporting introductions from Interior Alaska to the Susitna basin and from Western Alaska to the Kenai Peninsula. Third, we confirm that a long presumed postglacial relict population of pike near Yakutat Alaska is indeed native and genetically unique from other Alaska populations. Taken as a whole, these results shed new light on the history and current state of this persistent and on‐going invasion of a genetically depauperate apex predator.

By harnessing advances of modern genetics, we were able to detect population structure in non‐native populations despite low species‐level diversity and evidence of reductions in diversity consistent with bottlenecks. Broadly, introduced populations were split into three genetic groups: two in the Susitna River/Yentna River drainages and waterbodies of the Knik Arm and one on the Kenai Peninsula (Figures 4, 5, 6, 7). Introduced populations in the Susitna River/Yentna river and Knik Arm were split into two genetic clusters, Southcentral I & II, (see Figures 4 and 5), and confirmed via phylogenetic inference (Figure 7). This divergence is obvious when *K* = 4 and is maintained at higher estimates of *K*. These clusters were composed of Alexander Creek, Alexander Lake, Deshka River, Otter Lake, Shell Lake (Southcentral I), and Anderson Lake, Bulchitna Lake, Tukallah Lake, and Yentna River (Southcentral II). While some of these groupings conform with expectations of geographic and hydrologic connectivity (e.g., Alexander Creek flows out of Alexander Lake), others do not (Figure 6). For example, Bulchitna Lake (Southcentral II), the putative first site of pike introduction south of the Alaska range mountains, is physically close to both Alexander Creek and Shell Lake (Southcentral I) but appears to be genetically distinct. It is not clear to what extent the genetic distinctiveness of Bulchitna Lake reflects unknown idiosyncratic aspects of the local site rather than restricted gene flow. It is notable, however, that the outlet of Bulchitna Lake has a make‐shift unmaintained dam that likely reduces gene flow between the lake and nearby populations, while other sites do not have obvious barriers to dispersal (Jalbert, 2018).

Despite lingering uncertainty regarding the origins of non‐native populations, admixture plots in Figure 4 shed some light on allele sharing with native populations. For example, Southcentral I contain shared alleles with Fairbanks, Lake Clark, and Lake Nerka. This is also shown in F_ST_ estimates, which are lower for comparisons between Fairbanks and the above populations (0.239–0.267) than either Fairbanks and the rest of the introduced populations (0.335–0.339) or the Southcentral I group compared to Yukon Flats and Minto Flats (0.321–0.335). By contrast, the Anderson Lake, Bulchitna Lake, Tukallah Lake, and Yentna River populations (Southcentral II) shared alleles with Yukon Flats and Minto Flats as well as a reduced F_ST_ (0.193–0.204) compared to other native populations. One possibility is that the Southcentral I group was indeed founded by multiple source introductions from both Interior and Western Alaska (see also Figure S1). A nonmutually exclusive explanation is that gene flow and introgression from the Kenai Peninsula group that tends to share alleles with Western Alaska has created admixture to Southcentral I following initial human‐mediated introduction. Gene flow from the Kenai Peninsula to the Susitna would require human vectors or successful dispersal through brackish waters of Cook Inlet, which is likely possible given the known variation in salinity tolerance of the species (Sunde et al., 2018). While it is tempting to use these data to make definitive conclusions about the origins of the introduced populations, we advise caution for two reasons: (1) our sampling of native populations was far from exhaustive; thus, introductions may have resulted from unsampled “ghost populations” (Beerli, 2004); and (2) we lack contemporary samples from the time of introduction (1960s–2000s), which makes it difficult to determine with confidence the origin of the introduced populations as native populations have likely continued to evolve via both selection and drift during this time. This is especially likely in a species with limited gene flow and low levels of nucleotide diversity. This low level of gene flow is exemplified by investigating pike from Alexander Creek and its headwater, Alexander Lake. Despite close geographic proximity, pike in Alexander Creek exhibited more private alleles and higher estimates of 𝝅 than pike in its headwater lake, Alexander Lake (Susitna, Alaska, USA), and measures of differentiation between the groups were not zero (F_ST_ = 0.047 ± 0.011).

In contrast to the Southcentral I and II groupings, the two sites on the Kenai Peninsula most likely originated via introductions from Western Alaska. Genome‐wide F_ST_ estimates were high between the Kenai Peninsula samples and the other populations (both introduced and native) but lower between Stormy Lake and the two sampled Western Alaska populations (Lake Clark and Lake Nerka; 0.224 and 0.199, respectively). A Western Alaska origin for the Kenai Peninsula samples was supported by both population genetic and phylogenetic analyses, indicating that either these lakes or other lakes in the vicinity of Lakes Clark and Nerka were the origins of pike in the Kenai Peninsula. The first documented recovery of pike in Stormy Lake was in 1970, providing several decades for pike to disperse, but movement between Stormy Lake and Tiny Lake would involve dispersal through drainage basins and crossing of a saltwater barrier, which as discussed previously is unlikely for this scenario. The low likelihood of dispersal by pike without assistance between Stormy Lake and Tiny Lake paired with evidence of admixture within Stormy Lake (Figure 4) and higher nucleotide diversity than most introduced populations suggest that translocations within and/or multiple introduction events to the Kenai Peninsula have occurred. Our results suggest that the Kenai Peninsula pike originated from multiple sources from Western Alaska, and, at least in Stormy Lake, those founding sources were genetically distinct from populations in Interior Alaska. Although the vectors of introduction are not known, it is most plausible that intentional introductions with the use of floatplanes given their widespread use in Alaska and that all sites are within flight distances of each other. Indeed, private floatplanes are increasingly implicated in the introduction of aquatic invasive species in Alaska (Schwoerer et al., 2022).

The origin of pike in the Antlen River in the Yakutat region of Southeast Alaska has long been suspected to be a postglacial artifact but has remained unclear. Our genetic data from the Antlen River strongly suggest a native origin based on high genome‐wide pairwise F_ST_ between all populations studied and phylogenetic analyses which place Yakutat pike as distantly related to all other Alaska pike. In addition, nucleotide diversity of Antlen River pike exceeds that of numerous introduced populations (Figure 2a) and Tajima's *D* indicates a relatively recent population bottleneck. While it is unclear if Antlen River pike are survivors from a glacial refugium, it is clear that they did not serve as the origins for the introduced populations in Southcentral Alaska (F_ST_ range 0.401–0.419), nor are they closely related to other native Alaska populations. Population genetic and phylogenetic analyses of existing samples indicate the closest relationship of the Yakutat pike is to samples from Eagle Lake in Ontario, Canada, but again the effect of “ghost populations” is likely. Based on geography and the high F_ST_ values between the Yakutat pike and native Alaska populations, we believe it is likely that Yakutat was colonized by pike with a similar genetic background to pike that colonized east of the North American Continental Divide. Due to low sample size for the Eagle Lake pike from Ontario (*n* = 5), we did not perform F_ST_ or admixture analyses on these samples. Clearly, more sampling east of the North American Continental Divide and in other drainages geographically intermediate to Yakutat and those examined in this study is needed to further illuminate the relationship of Yakutat area pike in the Antlen River.

Our results contribute to a broader emerging story of pike as an increasingly successful global invader (Dunker et al., 2018). In the Colorado River, pike have been a conservation challenge since the 1970s (Tyus & Beard, 1990) and the recent invasion and spread of pike in tributaries of the Columbia River is a cause of widespread concern (Carim et al., 2019; Carim et al., 2022; Muhlfeld et al., 2008). Our findings, coupled with the observations of successful invasions elsewhere, make clear that pike are not limited, at least in the short term, by their lack of genetic diversity. Although it is unknown whether pike have adapted to novel environments through genetic or plastic mechanisms (Berghaus et al., 2019), the lack of diversity appears not to preclude genetic‐based adaptation in at least some other non‐native freshwater fishes (Koskinen et al., 2002). While it is possible that over the long‐term non‐native pike will decline or even exhibit local extinctions as other invasive species have been known to do (e.g., Cooling & Hoffmann, 2015), it seems equally possible that pike are not only here to stay, but likely to flourish in new habitats, especially in the light of widespread warming. Patterns from Europe serve as a cautionary tale, suggesting that co‐existence between salmonids and pike may only be possible where sufficient spatial and thermal refugia exist within habitats (Hein et al., 2014). In Alaska, it remains unclear how native fish communities will adapt to both the effects of ongoing pike invasions together with other ecological stressors in an era of rapid environmental change (Schoen et al., 2017; Westley et al., 2021).

## CONFLICT OF INTEREST STATEMENT

The authors have no competing interests to declare.

## Supporting information


Figure S1.

Table S1.

## Data Availability

Sequence data generated for this study have been deposited in the National Center for Biotechnology Information Sequence Read Archive under BioProject PRJNA949726.
