# Supplementary material for: Genomics reveal the origins and current structure of a genetically depauperate freshwater species in its introduced Alaskan range
Source: Evol Appl. 2023 May 12;16(6):1119–34. doi: 10.1111/eva.13556 (PMC10286226; doi:10.1111/eva.13556)
Supplement: Supplementary file 1 — Figure S1. [file EVA-16-1119-s002.docx]

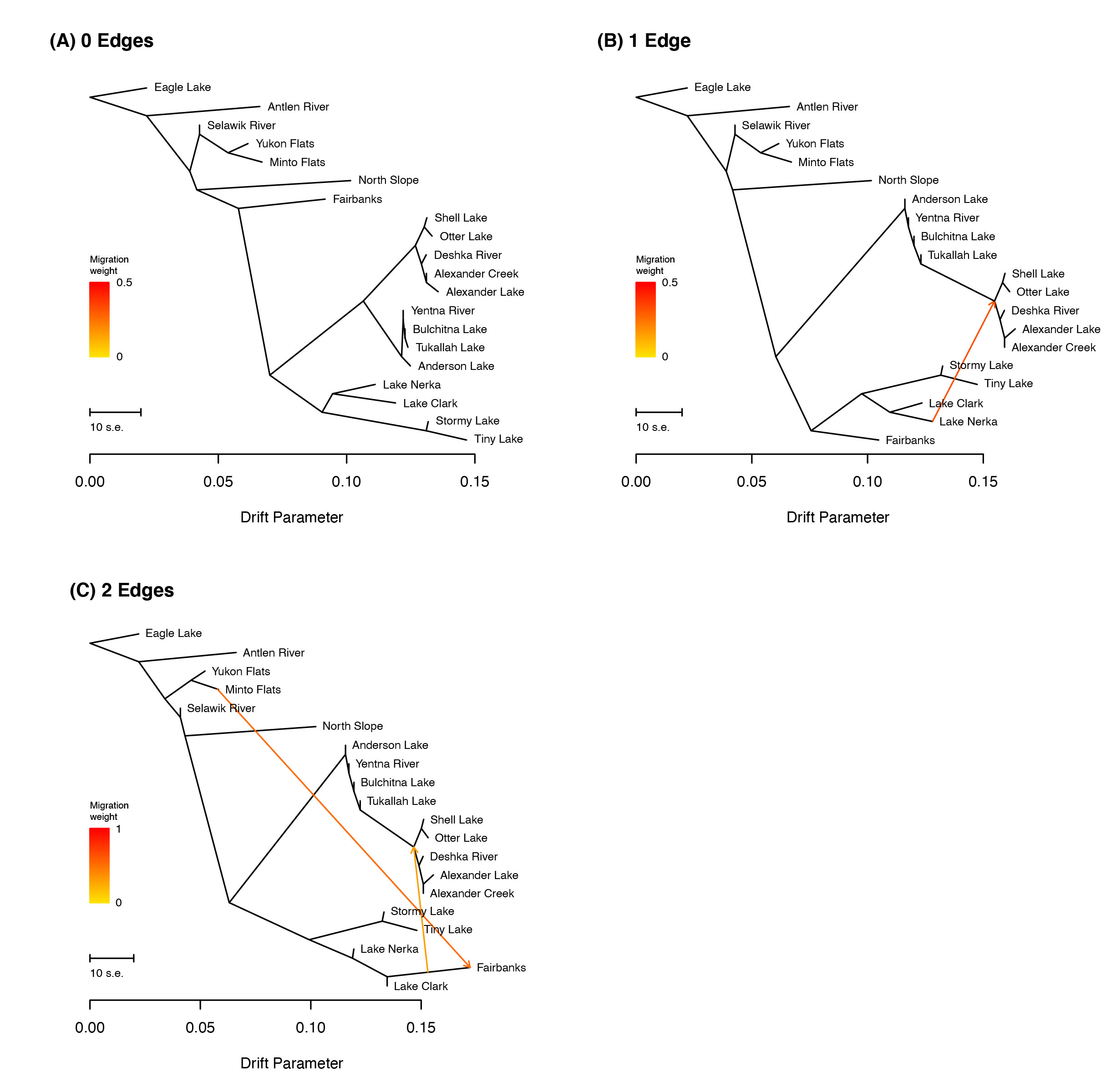


Supplemental Figure S1 TreeMix analysis of Alaskan pike. The 358 pike data set generated for phylogenetic analysis (compare to Figure 6) was analyzed with TreeMix v1.13 (Pickrell and Pritchard, 2012). Eagle Lake was specified as the outgroup and topologies generated with values of *m* migration edges 0, 1 and 2. As the SNPs were pruned, presumably unliked and SNP density was low in Alaskan pike, we specified -k 10 with TreeMix. The -global flag was included to conduct rearrangements after the initial tree construction.
